# A pilot study of nailfold capillaroscopy in hereditary transthyretin amyloidosis

**DOI:** 10.1038/s41598-022-15779-2

**Published:** 2022-07-09

**Authors:** Dayoung Kim, Jeeyoung Oh, Hong Ki Min, Hae-Rim Kim, Kyomin Choi

**Affiliations:** 1 Department of Neurology, Konkuk University Medical Center, Seoul, Republic of Korea; 2 Department of Neurology, Konkuk University Medical Center, Konkuk University School of Medicine, Seoul, Republic of Korea; 3 Division of Rheumatology, Department of Internal Medicine, Konkuk University Medical Center, Seoul, Republic of Korea; 4 Division of Rheumatology, Department of Internal Medicine, Konkuk University Medical Center, Konkuk University School of Medicine, Seoul, Republic of Korea

**Keywords:** Peripheral neuropathies, Systemic sclerosis, Diagnostic markers

## Abstract

Nailfold capillaroscopy (NFC) is a safe and non-invasive imaging tool for evaluating microvascular abnormalities. This retrospective cross-sectional study aimed to analyze the NFC outcomes and clinical characteristics in patients and an asymptomatic carrier with *transthyretin (TTR)* gene mutation. The participants consist of eight patients with genetically and clinically confirmed hereditary amyloidogenic transthyretin (ATTRv) amyloidosis and one asymptomatic carrier. The *TTR* gene mutant forms of six male and three female participants from six families were Asp38Ala (five patients), Lys35Asn (three patients), and Ala36Pro (one patient). All participants showed decreased capillary density, dilatated capillaries, and destructed architecture in NFC. Early progression identification of a carrier to patients with symptoms is a major concern from a therapeutic viewpoint in ATTRv amyloidosis. Therefore, further studies with a larger number of subjects will be needed to determine the use of NFC as an early detection tool.

## Introduction

Nailfold capillaroscopy (NFC) is a safe and non-invasive imaging tool for evaluating the microvascular abnormalities and capillary structure in the nailbed area. So far, NFC application mainly included rheumatic disease assessment and their clinical presentation, such as systemic sclerosis and Raynaud’s phenomenon^1^. Recently, NFC has been attempted on various diseases that are expected to have microcirculation pathology beyond rheumatic diseases. The most representative example related to these new diagnostic attempts is a diabetic complication, wherein patients with diabetes show reduced capillary length, irregular distribution, abnormal morphology, and reduced density compared with normal subjects in NFC^2,3^. Particularly, a positive correlation was found in the NFC score in diabetic retinopathy and polyneuropathy^3,4^.

Hereditary amyloidogenic transthyretin (ATTRv) amyloidosis is a rare, autosomal dominant, adult-onset systemic disease caused by a point mutation in the gene encodes transthyretin (TTR). TTR is a 55-kDa homotetramer protein that is largely synthesized by the liver and accounts for approximately 90% of TTR production. Additionally, TTR is also synthesized by retinal pigment epithelium cells and choroid plexuses of the brain^5^. Systemic and localized amyloid accumulation is related to the disease. Among them, the ocular involvement after liver transplant is observed although plasma TTR cannot cross the blood-retina barrier. Thus, ocular manifestations are largely due to the local TTR production^6^. Until 1990, ATTRv amyloidosis was considered an endemic rare disease that was observed in Portugal, Sweden, and Japan^7–9^. Since then, several endemic areas were additionally reported in a recent study that estimated the cumulative number of people with ATTRv amyloidosis in 10,186 persons, with a range of 5526–38,468^10^. The epidemiologic study of endemic areas and the incidence is likely to be updated with increasing disease awareness among clinicians and wider use of the genetic test.

One of the suggested pathomechanisms is that axon loss occurs due to microangiopathy as ATTRv progresses although the pathophysiology of neuropathy in ATTRv is unclear^5,11^. This argument is based on the pathological finding that endoneurial blood vessels are invaded and destroyed by amyloid deposition^12^. We evaluated whether NFC can support this view as an easy and non-invasive way to observe microcirculation abnormalities in patients with ATTRv. Additionally, this study aimed to identify the disease onset in carriers promptly through NFC and determine whether NFC can reflect disease progression in patients with ATTRv amyloidosis. This study reports the NFC outcomes in eight patients with ATTRv amyloidosis and one asymptomatic carrier. The NFC patterns of the participants were investigated, which reported an association between these patterns and the clinical features of ATTRv amyloidosis.

## Methods

### Participants and ethical statements

This retrospective cross-sectional study included eight patients who were genetically and clinically confirmed with ATTRv amyloidosis and one asymptomatic carrier. The medical records of participants at a single tertiary medical center from April 2017 to December 2020 were investigated. Exclusion criteria were the presence of preexisting diseases, such as systemic connective tissue diseases or diabetes that affect the vascular architecture, or a history of trauma to the periungual fold of the fingers. This study was approved by the Institutional Review Board of Konkuk University Medical Center (KUMC-IRB), Seoul, Korea (No. 2021-04-046), and informed consent was exempted under the supervision of KUMC-IRB in consideration of the retrospective characteristics of this study, which is limited to medical record reviews that do not contain personal identification information. This study conformed to the ethical norms and standards of the Declaration of Helsinki.

### Investigation of clinical manifestations

Clinical data were collected from available medical records, consisting of age at onset, medical history, family history, the genotype of TTR mutation, clinical manifestations, and adjusted treatment. The main clinical presentation of each patient with ATTRv amyloidosis was classified as neuropathy, cardiomyopathy, or mixed types, and all participants were subjected to a series of related tests during initial diagnosis^13^. They underwent echocardiography and Holter monitoring to confirm amyloidogenic cardiomyopathy. Additionally, a nerve conduction study and autonomic nervous function screening test (heart rate variability, Valsalva maneuver, and tilt-table test) were performed to evaluate amyloidogenic peripheral neuropathy. Carpal tunnel syndrome (CTS), among neuropathy, was determined by clinical criteria and supplementary diagnostic methods^14^. Clinical criteria consist of compatible neuropathic pain, Tinel’s sign, or Phalen’s maneuver before the occurrence of lower extremity abnormalities. Disproportional solitary damage of the median nerve in the supplementary methods was confirmed by nerve conduction study or ultrasonography^15,16^. Neuropathy severity of patients with ATTRv amyloidosis was classified by Polyneuropathy disability (PND) scoring system^17^. This system includes the following five stages: stage 0, no nerve impairment and visible symptoms; stage 1, a sensory disturbance in the lower extremities but the walking capacity of the patient is not affected; stage 2, the patient might have some difficulty in walking but usually does not require walking aids, such as sticks or crutches; stage 3a, the patient might require support from a single walking stick or crutch; stage 3b, the patient might require support from two sticks or crutches, and stage 4, the patient is wheelchair bounded or bedridden. All participants had valid medical records that confirm the retinal capillary and vitreous structure status to assess ocular symptoms of amyloidosis.

### Nailfold capillaroscopy

The NFC test was performed by a skilled examiner who was blinded to the patients’ clinical amyloidosis status. Each patient was acclimatized for 20 min at a room temperature of 24 °C before the NFC examination. The examiner used a drop of immersion oil to increase the visibility of the microvessels in the nailfolds. The capillaries on the left and the right four fingers, except the thumb, were observed with a microscope (× 100 and × 400 magnifications; Olympus SZ-PT, Olympus, Tokyo, Japan), and photographs were taken of the last distal row of capillaries using a digital camera (Polaroid, Minnetonka, MN, USA). Four images were taken from eight fingers (2 of each finger at each magnification), and the evaluation was performed in a total of 32 fields.

The recently described semiquantitative rating scale (0: no changes; 1: < 33% of capillary alterations/reduction; 2: 33%–66% of capillary alterations/reduction; and 3: > 66% of capillary alterations/reduction) was adopted^1,18^. The following six capillaroscopic parameters were obtained: capillary density, dilated capillaries, giant capillaries, hemorrhage, ramification, and architectures. Normal nailfold capillaries consist of one loop with a diameter of < 20 μm. Meanwhile, ramified capillaries show patterns of bushes by additional several coils that originate from normal capillaries. The nailfold capillary architecture indicates the vessel arrangement consistency. The cut-off value for decreased nailfold capillary density was an average of nine uniform vascular loops per 1 mm in fingers. The alignment is lost, and the shape of the loops cannot be distinguished under pathological conditions. Each parameter was scored by a semiquantitative rating system and measured on each finger, and an average score of the eight fingers was recorded. The total score is calculated by adding up their averages. The lowest score means close to the normal condition, and a higher score indicates a distorted microvascular limb status.

## Results

### Study population and clinical characteristics

The study examined eight patients and one asymptomatic carrier in six families with pathogenic *TTR* gene mutation (Table 1). Of them, six were males and three were females with a mean age of 54 years (range 44–64 years). All participants were Koreans of East Asian descent. The mean disease duration of ATTRv amyloidosis was 7.5 years (range 5–11 years) upon NFC testing in 8 patients. Three mutation types of TTR gene were included, namely Asp38Ala (five patients), Lys35Asn (three patients), and Ala36Pro (one patient). Patient No. 3 had a neuropathy type amyloidosis, and the remaining seven patients were mixed type. Patients No. 1 and No. 6 showed vitreous opacity due to amyloid deposits without retinal vessel change. All eight patients had peripheral neuropathy, and five of them had additional neuropathology limited to the hand called CTS. Among the autonomic disorders, orthostatic hypotension occurred in all eight patients, and intractable diarrhea was repeated in six patients. Additionally, two patients and one asymptomatic carrier were smokers during the NFC testing. Except for the asymptomatic carrier, all eight enrolled patients were treated with transthyretin stabilizer (tafamidis) or small interfering RNAs (siRNAs) targeting the *TTR* gene (patisiran).

**Table 1:** Clinical characteristics and nailfold capillarography of participants.

Patient no	Family no	Sex	Age	Smoking hx	ATTRv characteristics								Nailfold capillarography*						
					Disease duration, (yr)	Applied treatment and duration (medication, yr)	Mutation	Main clinical presentation	PND score	Ocular manifestation	CTS	Autonomic dysfunction	Capillary density	Dilated capillaries	Giant capillaries	Hemo-rrhage	Ramifi-cation	Archi -tecture	Total sum score
1	F1	F	53	−	11	Patisiran, 6	Lys35Asn	Mixed	3b	Vitreous opacity,	+	OH, diarrhea	0.625	0.625	0	0	0	0.750	2.000
2	F1	M	49	−	6	Tafamidis, 3	Lys35Asn	Mixed	1	None	−	OH, diarrhea	0.875	1.125	0	0	0	0.750	2.750
3	F2	F	59	+	4	Tafamidis, 1	Lys35Asn	Neuropathy	1	None	+	OH	0.750	1.500	0	0	0	0.875	3.125
4	F3	F	64	−	10	Patisiran, 6	Asp38Ala	Mixed	2	Vitreous opacity,	+	OH, diarrhea	0.875	1.000	0	0	0	0.625	2.500
5	F3	M	60	−	6	Tafamidis,1 then switch to Patisiran, 2	Asp38Ala	Mixed	1	None	+	OH, diarrhea	0.625	0.750	0	0	0	1.250	2.625
6	F3	M	44	−	(−)	–	Asp38Ala	Asymptomatic carrier	0	None	−	None	3.000	0	0	0.125	0	3.000	6.125
7	F4	M	51	+	8	Tafamidis,1 then switch to Patisiran, 2	Ala36Pro	Mixed	2	None	+	OH	0.750	1.125	0	0	0	1.625	3.500
8	F5	M	59	−	9	Patisiran, 1	Asp38Ala	Mixed	3a	None	−	OH, diarrhea	1.500	1.25	0	0	0	2.500	5.250
9	F6	M	55	+	5	Patisiran, 4	Asp38Ala	Mixed	1	None	−	OH, diarrhea	0.250	1.000	0	0	0	1.500	2.750

*PND* polyneuropathy disability, *CTS* carpal tunnel syndrome, *OH* orthostatic hypotension; * 6 items were measured with three scales on each finger and the average score of the eight fingers was recorded. (Scale 0: no changes; 1: < 33% of capillary alterations/reduction; 2: 33%–66% of capillary alterations/reduction; and 3: > 66% of capillary alterations/reduction).

### NFC measurement

**Figure 1:**
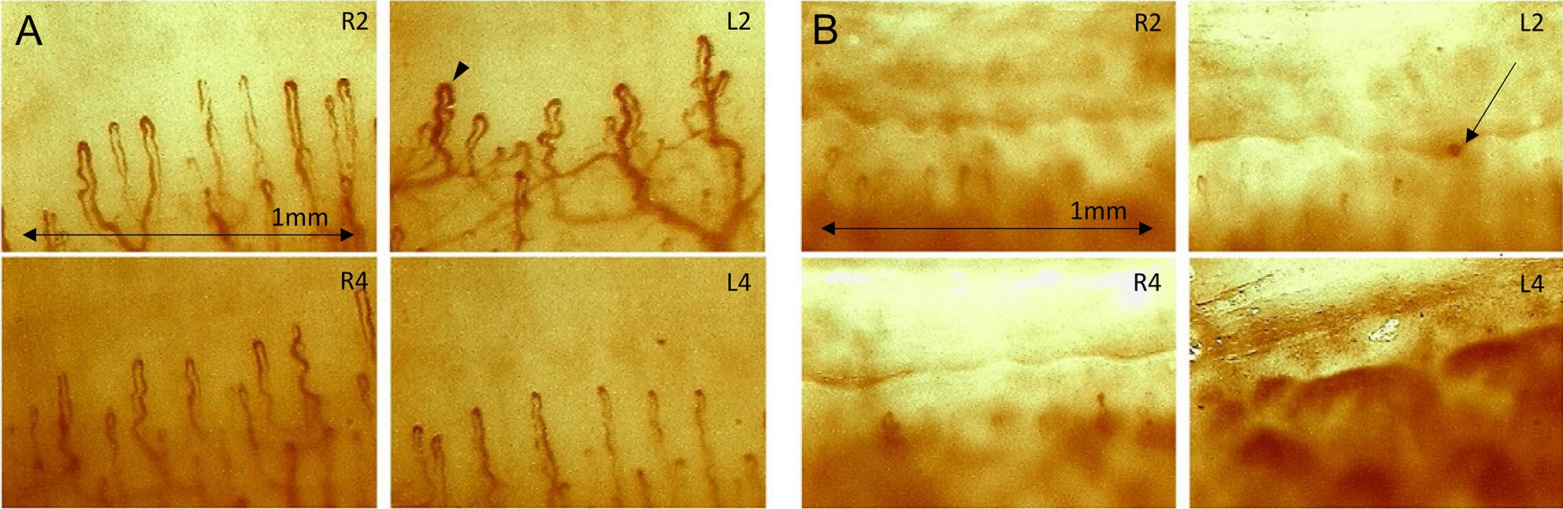
Patient No. 1 showed slightly decreased density and dilatation (arrowhead) of capillaries (**A**), and Patient No. 6 had few capillaries because of extensive architecture damage, and hemorrhage (arrow) in the nailbed area (**B**). These were taken from the bilateral second and fourth fingers. (× 400 magnifications; Olympus SZ-PT, Olympus, Tokyo, Japan; R: right, L: left, 2: second finger, 4: fourth finger.

All eight patients and one asymptomatic carrier showed abnormalities in the components of capillary density, dilatated capillaries, and architecture (Table 1). The mean of the 9 participants’ total score was 3.403 (range 2.000–6.125). Patient No. 1 with the longest duration of illness and severe neuropathic symptoms (PND score of 3b) among all participants had the lowest, near-normal result in the total score (Fig. 1A). Two patients with ocular amyloid pathology due to localized TTR production (Patients No. 1 and 4) had relatively low scores among the subjects with total scores of 2.000 and 2.500. The highest score was 3.500 in the five patients with CTS. The asymptomatic carrier (Patient No. 6) showed the lowest capillary density or highest avascular areas in NFC and had the highest score of 6.125 (Fig. 1B). Two (Patients No. 6 and 8) of the three smokers had high scores (6.125 and 5.250, respectively), wherein one was an asymptomatic carrier and the other had a severe status with a PND score of 3a. The NFC result for each type of *TTR* gene mutation, excluding carriers, revealed two patients (Patient No. 1 and 4) with the longest disease duration (Lys35Asn and Asp38Ala, respectively) with the lowest scores (2.000 and 2.500, respectively) in each group.

## Discussion

Microangiopathy caused by blood-nerve-barrier disruption and extracellular amyloid is considered one of the major pathophysiologies in ATTRv amyloidosis^5^. Retinal and choroidal microvasculature destruction due to localized amyloid deposit has also been reported with ATTRv amyloidosis, which has been claimed as the main evidence that suggests clinical microangiopathy^19^. Another key feature of ATTRv is autonomic neuropathy, in which small fiber damage is prominent. Selective and preferential A-delta and C fiber damage before extensive systemic abnormalities appear responsible for these features^20^. In a previous case report, Raynaud's phenomenon is one of the autonomic dysfunctions of the microvasculature and has been reported as one of the major clinical phenotypes of ATTRv^21^. NFC results can be expected to be abnormal in patients with ATTRv because of the presence of microvascular pathology and autonomic nervous system dysfunction in ATTRv. However, the pattern or findings of NFC reflecting microvasculature and autonomic nervous system in patients with ATTRv amyloidosis has never been described yet.

All eight patients with ATTRv amyloidosis and one asymptomatic carrier showed decreased capillary density, dilatated capillaries, and destructed architecture although differences in severity were seen in this study. Nevertheless, interestingly, a participant with asymptomatic carrier status had the most severe form of impairment, and patients with a long disease duration showed relatively good NFC outcomes. Hence, it can be inferred that risk factors, other than *TTR* gene mutation, affect NFC results, and smoking is likely considered a risk factor in this study. Smoking is one of the main risk factors for endothelial damage, and asymptomatic smokers have been reported to be more commonly accompanied by NFC abnormalities than nonsmokers^22^. However, how smoking affects ATTRv amyloidosis or how smoking negatively affects NFC in the presence of comorbid underlying medical conditions has not been established. Therefore, additional studies comparing the NFC results by classifying carriers with *TTR* gene abnormalities as smokers and nonsmokers would be recommended. Another factor in this outcome may be the effect of adjusted treatment. Two patients with the longest disease duration also received siRNA treatment for the longest duration among patients (Table 1). The microvascular damage is uncertain to have a reversible course in this study, assuming that ATTRv and microangiopathy are related. Additionally, confirming the treatment effect on their good NFC test results is impossible. However, this suggests that subsequent studies should consider the treatment effects, as well as the association of ATTRv and NFC results.

This is the first report to apply NFC as an evaluation tool for ATTRv amyloidosis. NFC abnormalities may have been presented to accompany the group with *TTR* gene mutation, but significant limitations need to be considered. First, the number of evaluated subjects was very small because ATTRv amyloidosis is a very rare disease. A previous study that analyzed NFC results with systemic diseases other than rheumatic diseases demonstrated the association between the longer period and severity of diabetes mellitus status and more severe capillary damage measured by NFC^23^. Additionally, NFC abnormalities were confirmed to be more prominent in patients with glaucoma and essential hypertension than in the non-disease group^23^. Whereas, the disease duration, clinical severity, and NFC results did not show proportional results in this study, which is likely due to the small number of participants. Second, from a viewpoint related to the ATTRv research, surveying the relationship between genotype and phenotype in *TTR* gene mutation is important, but such an analysis was not possible in this study. Third, as a cross-sectional study, the time course of the disease and co-risk factors were not adequately adjusted. Particularly, the peripheral neuropathy status of the upper extremity of the participants would all vary depending on the disease duration, the patient’s underlying condition, and treatment experience. All patients’ severity of polyneuropathy and carpal tunnel syndrome was not measured in detail. A follow-up study is needed that considering the specific NFC results obtained in the group in which the disease does not start in the upper extremity even with polyneuropathy. Additionally, a separate evaluation is estimated to be necessary for patients with only CTS among patients with ATTRv. Fourth, the NFC assessment method should be applied differently depending on the disease although we applied the standard method in rheumatoid disease and diabetes. However, this limitation is expected to be improved as further studies are conducted.

From the therapeutic perspective of ATTRv amyloidosis, the current greatest interest is finding a suitable group for early treatment because early treatment is effective in preventing irreversible damage and poor quality of life^5^. However, early ATTRv diagnosis is difficult due to disease rarity. Additionally, the criteria for determining the onset and treatment initiation may be ambiguous even in a group that monitors the disease onset due to family history. Therefore, the authors wanted to investigate the effectiveness of NFC as an applicable and simple test method considering the pathophysiology of ATTRv. Hence, this pilot study suggests conducting additional larger and well-controlled studies to verify the possibility of NFC as an early diagnostic tool in asymptomatic carriers and patients with ATTRv amyloidosis.
